# *In vivo* third-harmonic generation microscopy study on vitiligo patients

**DOI:** 10.1117/1.JBO.25.1.014504

**Published:** 2019-11-28

**Authors:** Yi-Hua Liao, Yu-Hsiang Su, Yuan-Ta Shih, Wen-Shiang Chen, Shiou-Hwa Jee, Chi-Kuang Sun

**Affiliations:** a National Taiwan University Hospital, National Taiwan University College of Medicine, Department of Dermatology, Taipei, Taiwan; b National Taiwan University, Graduate Institute of Photonics and Optoelectronics, Department of Electrical Engineering, Taipei, Taiwan; c National Taiwan University Hospital, National Taiwan University College of Medicine, Department of Physical Medicine and Rehabilitation, Taipei, Taiwan; d Cathay General Hospital, Department of Dermatology, Taipei, Taiwan

**Keywords:** basal keratinocytes, third-harmonic generation, harmonic generation microscopy, melanin, melanocytes, skin, vitiligo

## Abstract

Melanin is known to provide strong third-harmonic generation (THG) contrast in human skin. With a high concentration in basal cell cytoplasm, THG contrast provided by melanin overshadows other THG sources in human skin studies. For better understanding of the THG signals in keratinocytes without the influence of melanin, an *in vivo* THG microscopy (THGM) study was first conducted on vitiliginous skin. As a result, the THG-brightness ratio between the melanin-lacking cytoplasm of basal cells and collagen fibers is about 1.106 at the dermal–epidermal junctions of vitiliginous skin, indicating high sensitivity of THGM for the presence of melanin. We further applied the *in vivo* THGM to assist evaluating the therapeutic outcome from the histopathological point of view for those showed no improvement under narrowband ultraviolet B therapy based on the seven-point Physician Global Assessment score. Our clinical study indicates the high potential of THGM to assist the histopathological assessment of the therapeutic efficacy of vitiligo treatments.

## 1 Introduction

Vitiligo is an acquired chronic depigmentation disorder resulting from progressive loss of epidermal melanocytes and is clinically characterized by well-defined, white macules and confluent patches.^1^ The prevalence rate of vitiligo is around 1.0% worldwide,^1^ and the disfigurement leads to a negative impact on patients in terms of quality of life.^2^ The depigmentation in vitiligo is caused by the selective destruction of l-3,4-dihydroxyphenylalanine (DOPA)-positive melanocytes in the epidermis and sometimes those in the bulb and infundibulum of the hair follicles, sparing the DOPA-negative, nondendritic melanocytes in the middle/lower portions of the outer root sheath (ORS).^3^ The precise reason for melanocyte destruction remains unknown, but autoimmune, cytotoxic, neurohumoral factors, oxidative stress, and melanocytorrhagy have been proposed to explain the pathogenesis of vitiligo.^1^ The two major melanocyte reservoirs for repigmentation of vitiligo skin are the ORS or bulge area of the hair follicle and the perilesional epidermis.^4^ Ultraviolet (UV) radiation, including UVB or photochemotherapy (psoralen plus UVA), and excimer laser/light^3^^,^^5^^,^^6^ can stimulate the activation, proliferation, and migration of melanocytes from hair follicles and perilesional skin into the vitiliginous epidermis, followed by functional maturation to melanin-producing melanocytes, which result in perifollicular and marginal repigmentation in vitiligo, respectively.^4^ Vitiligo usually requires a long course treatment to observe skin repigmentation clinically. It is thus highly desired to find a noninvasive tool to provide early histopathological assessment for efficacy of various therapies used in vitiligo. It will be helpful to guide physicians through disease management.

Melanin is known to provide strong third-harmonic generation (THG) contrast in human skin.^7^^–^^9^ A recent study^10^ has further indicated the capability of noninvasive label-free THGM to differentiate melanocytes from Langerhans cells, which is not feasible in other *in vivo* label-free clinical-imaging modalities. This characteristic makes THGM ideal for the assessment of the vitiligo therapy and, with its high three-dimensional (3-D) resolution, to facilitate the understanding of the biologic mechanisms of repigmentation in vitiligo. In this study, to better understand the signal intensity of THG in vitiliginous keratinocytes with the minimal influence of melanin, an *in vivo* THGM study was conducted on vitiliginous skin, with a focus on the comparison of THG intensity between the cytoplasm of basal cells and collagen fibers. Stacks of images were acquired at vitiliginous skin. As a result of the analysis, the THG-brightness ratio between the melanin-lacking cytoplasm of basal cells to collagen fibers in vitiliginous lesions was measured to be 1.106 at the dermal–epidermal junction of vitiliginous skin. With this low ratio, indicating the high THG sensitivity of melanin, we further applied the *in vivo* THGM to assist evaluating the therapeutic outcome from the histopathological point of view for those showed no improvement under narrowband (NB)-UVB therapy based on the seven-point Physician Global Assessment (PGA) score. Among the patients without clinical improvement of their target vitiliginous lesions, nearly half of them showed significant THG features on the reappearance of melanocytes or increased melanin-containing basal cell distribution, which was not assessable by the traditional scoring based on the outlook of the lesions. Our study indicates the high potential of THG microscopy for the early and sensitive assessment of vitiligo therapy.

## 2 Clinical Study on the Basal Cell Cytoplasm to Collagen Fiber THG-Brightness Ratio

To diagnose skin conditions and diseases, the histopathological examination of excised tissues is the gold standard. However, together with a lengthy procedure, the painful biopsy process is associated with possible side effects like bleeding, infections, and scar formation. A least-invasive imaging tool, which can perform high-resolution virtual biopsy examination, is thus highly desired. Recently, optical imaging techniques, such as reflection confocal microscopy,^11^ optical coherent tomography,^12^ two-photon fluorescence microscopy,^13^ and THGM,^14^ have been widely applied for high-resolution *in vivo* skin imaging. With the excitation wavelength located at the 1300-nm high penetration window and a cubic nonlinearity for superior signal-to-background ratio imaging at deep tissues, THGM provides slide-free and label-free clinical imaging in human skin with a high 3-D resolution.^7^^,^^8^ We first intended to know the THG-brightness ratio of basal cell cytoplasm to collagen fiber in human skin without melanin influences. Image stacks consisting of multiple subimages representing different depths in human skin with vitiligo were thus acquired and analyzed. For a single patient, different stacks record images from different locations in the vitiligo lesion.

### 2.1 Harmonic Generation Microscope and Cr:Forsterite Laser

**Fig. 1:**
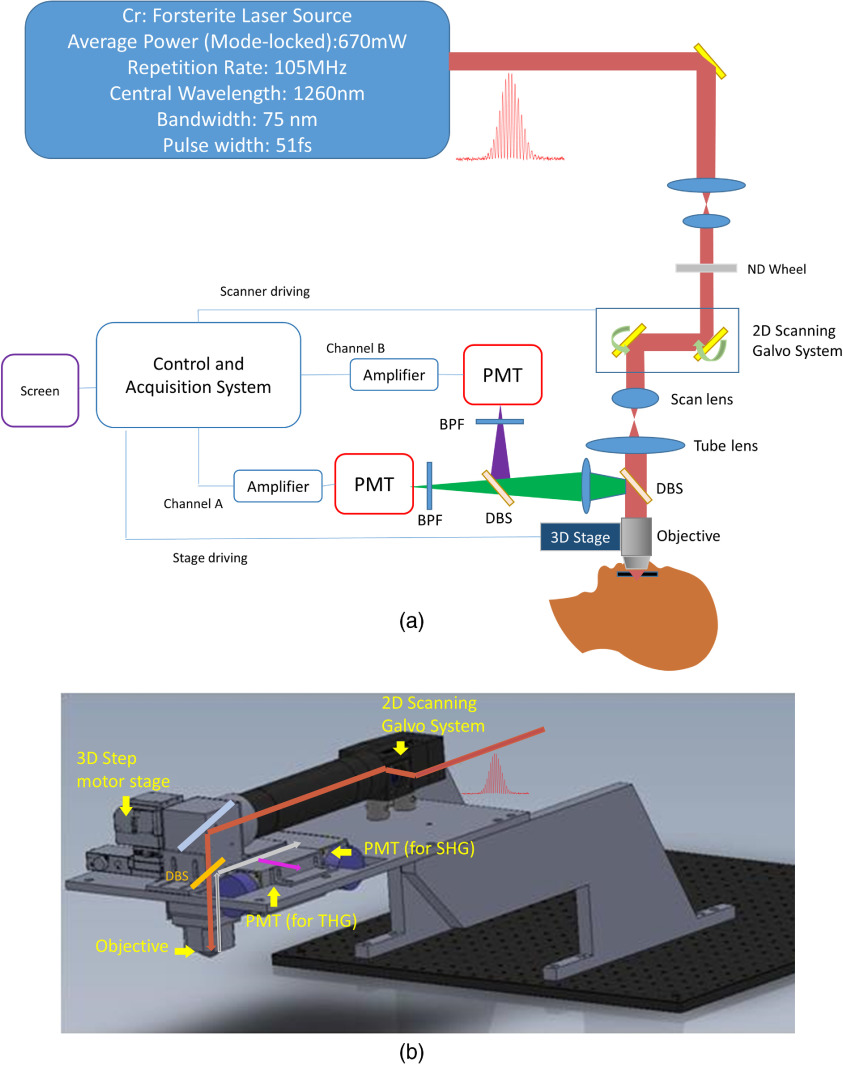
(a) A schematic diagram showing the system setup of the HGM used in the clinical trial. (b) The 3-D model of the HGM system. The optical path and important devices are shown. DBS, dichroic beam splitter; BPF, band-pass filter; PMT, phtotomultiplier tube; SHG, second-harmonic generation; THG, third-harmonic generation.

Considering the *in-vivo* clinical experiment on skin, we designed a vertical harmonic generation microscope (HGM) with a Cr:forsterite (Cr:F) laser for second-harmonic generation (SHG) and THG signals. The center wavelength of the Cr:F laser was around 1260 nm, and the average output power after objective was about 100 mW with a repetition rate of 105 MHz. The bandwidth of the Cr:F laser was improved to 75 nm and the pulse width was shortened to 51 fs for improved nonlinear excitation efficiency. As shown in Fig. 1(a), the Cr:F laser beam was collimated by a telescope that reduced the beam size at the same time. The beam, after passing through a neutral density wheel to attenuate the power to a safe level for clinical studies, was then guided into a galvoresonant scanning head (Thorlabs Laser Scanning Essentials Kit) to perform high-speed 2-D scanning. The scanning pattern was focused onto human skin by a water immersion objective with a numerical aperture (NA) of 1.15 (Olympus, UApoN340). Epi-SHG and epi-THG signals were collected by the same objective and reflected by a dichroic beam splitter (DBS). THG (around 420 nm) and SHG (630 nm) signals were divided by another DBS and sent to two individual photomultiplier tubes (PMTs; Hamamatsu R928P) with band-pass filters inserted (FF02-617/73 for SHG and FF01-417/60 for THG, both from Semrock). The objective was attached to a 3-D step motor so the position of the objective can be adjusted by both manual tuning and remote electrical control. The imaging plane (or the plane of observation) can be moved to different depths by tuning the 3-D stage along the optical axis. The remote electrical control was assisted by LabVIEW programs. We installed a motor-driven bed for conveniently adjusting the height of patients to reach the objective and to rapidly adjust the position of observation region. Figure 1(b) shows a realistic design of the system.

**Fig. 2:**
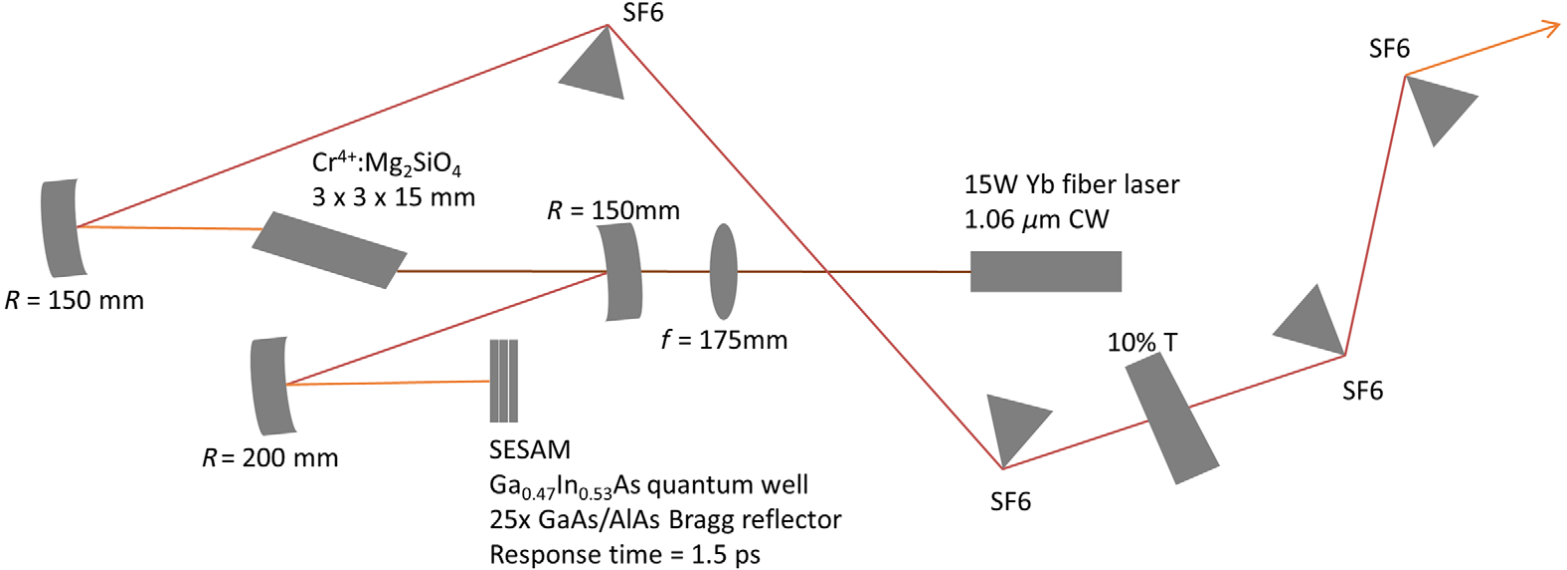
Schematic illustration of the Cr:F laser oscillator.

Figure 2 shows the design of the home-built Cr:F laser oscillator. The pump source was a linear-polarized single-mode continuous-wave Ytterbium fiber laser (YLR-15-1064-LP IPG), whose central wavelength was 1064 nm with 1-nm linewidth. The polarization was set to be horizontal for minimal reflection at the Brewster’s angle of the crystal. The pump beam was focused on the Cr:F crystal via a lens of 175-mm focal length, contributing a beam waist about 60  μm at the center of the crystal. The typical pump power was around 14.20 W for stable mode-locking and would be slightly adjusted according to the room temperature and humidity. The dimension of the Brewster-cut Cr:forsterite crystal was $3\times 3\times 15\;{\mathrm{mm}}^{3}$. The laser beam traveled along the a axis (15 mm) of the crystal and its polarization relied on the b axis of the crystal. The refractive index of the crystal is about 1.63, corresponding to the Brewster’s angle of 58.5 deg. The surface temperature of the crystal was kept at 5.0°C by a thermoelectric cooler with a temperature feedback loop. The hot side of the thermoelectric cooler was contacted with circulating water, which was kept at 15.0°C by an immersion circulator (Thermo Scientific SC150-A25). Pure nitrogen was purged on the surface of crystal, preventing moisture condensation.

This laser oscillator is with a standard z-fold confocal cavity. There were two concave mirrors with radius of curvature R=150  mm, located beside the crystal. One end of the cavity was a wedge output coupler with 10% transmission. The other end of the cavity was a semiconductor saturable absorber mirror (SESAM). SESAM was mainly composed of two ${\mathrm{Ga}}_{0.47}{\mathrm{In}}_{0.53}\mathrm{As}$ quantum wells and a 25× GaAs/AlAs Bragg reflector mirror. The absorption of the quantum wells could be saturated by high-intensity pulses with a recovery time of 1.5 ps. Therefore, femtosecond pulses would be almost lossless while the continuous wave components are attenuated by absorption. As a result, with SESAM as a slow absorber, the laser mode-locking was self-starting and stabilized. There were two Brewster-cut SF6 prisms in the cavity providing negative group delay dispersion to compensate the material dispersion and the self-phase modulation inside the crystal. SF6 prisms were chosen for less total third-order dispersion.

### 2.2 Image Acquisition Conditions

*In vivo* HGM images were acquired at lesional normal skin located over anterior chest wall, forehead, back, abdomen, shoulder, and neck. *In vivo* epi-SHG and epi-THG signals were collected with acquisition time 0.357  s/image. Signals from two channels were then mapped into 14-bit grayscale images with the size of 512×512  pixels, corresponding to a field of view of $295\times 295\;\mu m^{2}$. Images of different depths from the skin surface were acquired every 1.8  μm along the optical axis with the help of the step motor to which the objective was attached. Afterward, the images can then be stacked into a single tiff file with multiple subimages while each subimage is an image for a certain depth in skin. Fourteen stacks of images from nine patients (six men and three women) with vitiligo were obtained and analyzed.

The study protocol was approved by the Ethics Committee of National Taiwan University Hospital (No. 201403043DINC) and Taiwan Food and Drug Administration (No. 2014-07-18).

### 2.3 Protocol on Obtaining Brightness Ratio

We established the following protocol with five phases in order to obtain the melanin-lacking basal cell cytoplasm to collagen fiber THG-brightness ratio.

#### 2.3.1 Previewing phase

We avoided analyzing stacks with the following characteristics at the depth of dermal–epidermal junction: (1) Images were so blurred (because of lateral movement of the subject) that the outlines of basal cells cannot be recognized and that having three qualified subimages to analyze is impossible; (2) similar to normal skin, THG-bright pieces indicating accumulation of melanin can be seen among basal cells; and (3) THG brightness of basal cells was over 1.6 times that of collagen fibers, which is the case in normal phototype I skin^8^ and therefore is reasonable to doubt that there is melanin in basal cells. Phototype I skin is the lightest skin type with melanin.

#### 2.3.2 Region of interest selecting phase

Due to the optical scanning mechanism, images deep in biotissues become dim around the edge of the scanning pattern. Electronic noise from PMTs results in random grayscale values at those dim and uniformly dark regions. We analyzed only pixels with sufficient SNR and thus defined a rectangular region of interest (ROI) on the basis of a simple threshold. For each stack, we applied the threshold of 1600 in the grayscale value on THG images at the depth of basal layers, because an SNR greater than 10 was desired and the mean of THG grayscale values at uniformly dark regions was ∼150. A rectangular ROI can then be depicted based on the result of thresholding. It is also advisable to use algorithms, such as the Otsu method to obtain a more exact threshold. Pixels in ROI are further analyzed.

#### 2.3.3 Screening phase

The images at the depth of dermal–epidermal junction with the following characteristics were qualified to be processed: (1) SHG signals of collagen fibers with intensity greater than 1600 occupied more than 25% of the area in ROI. (2) The collagen fibers were clear enough to see their orientation. (3) There were at least 10 distinguishable basal cells in the ROI. For a stack to be counted into final results, it must have three qualified subimages to calculate the stack’s averaged THG-brightness ratio.

#### 2.3.4 Processing phase

THG brightness extraction for collagen fibers and basal cell cytoplasm was included in this phase. The programs used in the phase and algorithmic frameworks of them were developed and detailed in previous studies.^15^^,^^16^

**Fig. 3:**
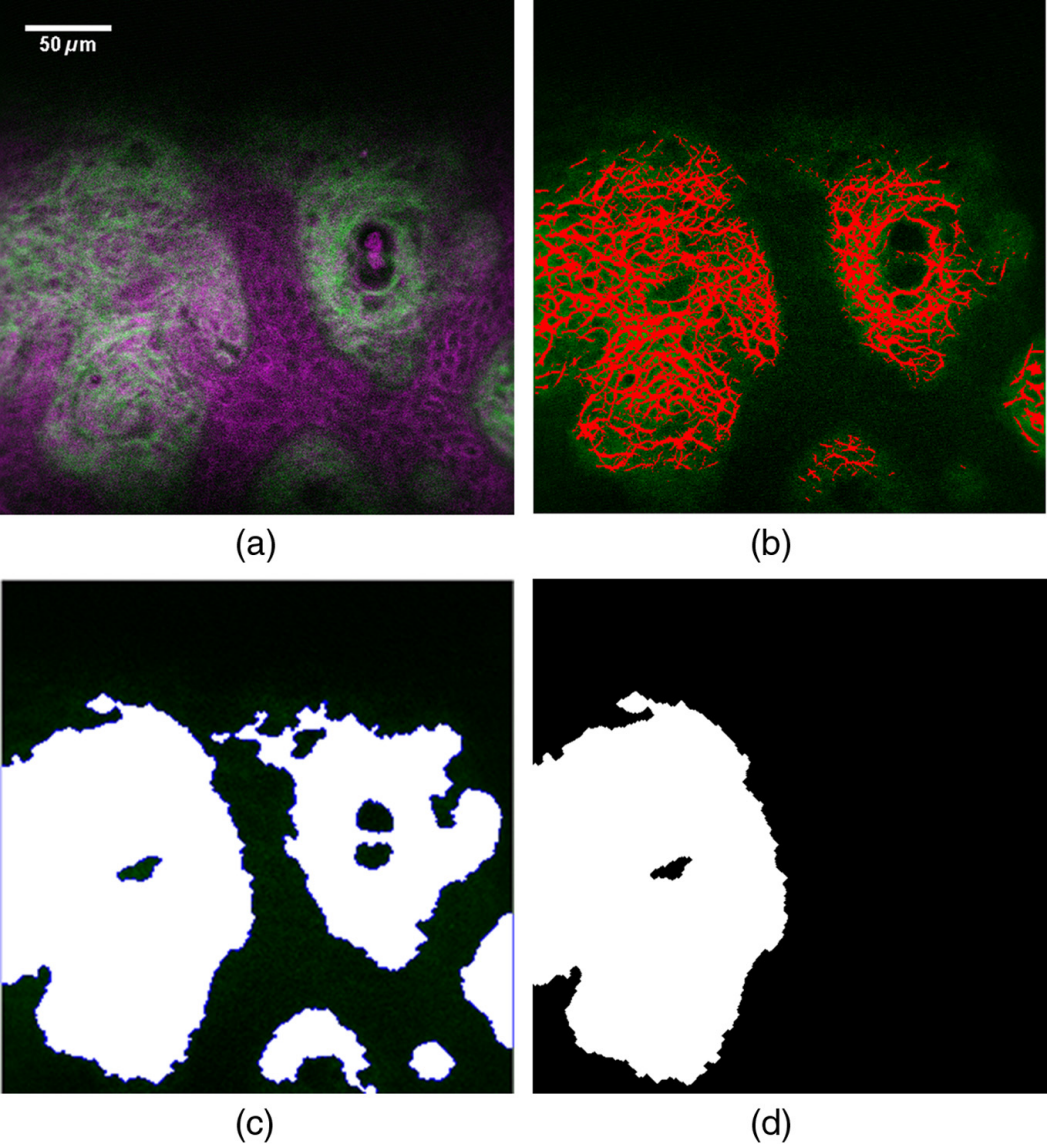
(a) *In vivo* THG and SHG microscopy images of vitiligo lesion with an *en face* view. Image was acquired at the depth of the dermal–epidermal junction. THG and SHG are shown in magenta and green pseudocolors. (b) Segmented collagen fiber is masked with red. (c) Initial ROI suggested by the program. (d) ROI was manually modified to avoid selecting capillary walls near fibers. Image size: $295\times 295\;\mu m^{2}$. Scale bar: 50  μm.

**Fig. 4:**
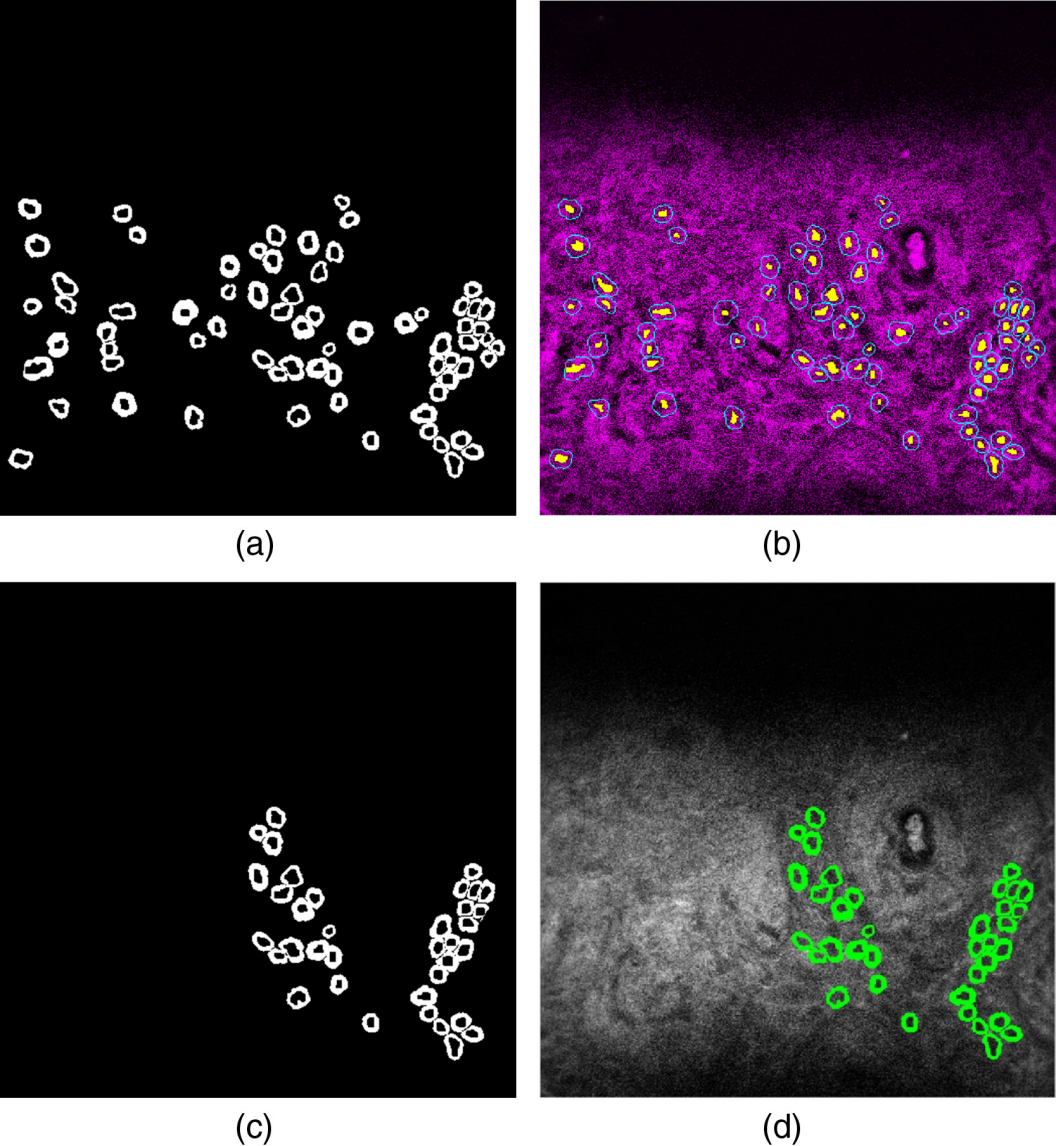
The same image, as shown in Fig. 3, was analyzed. (a) The initial cytoplasmic segmentation suggested by the program. (b) Imposition of the initial segmentation on the THG image. The bright blue boundaries delineate segmented cells. The segmented nuclei are masked yellow. (c) ROI is manually modified to avoid false segmentation and fiber regions. (d) Imposition of the modified segmentation on the THG image. Cytoplasmic regions are masked with bright green.

For the part of collagen fibers, at least three qualified subimages were put into the program to segment collagen fibers on the basis of SHG information (Fig. 3). The initial ROI suggested by the program was then partly removed if the region lied outside the rectangular ROI or covered THG signals obviously not induced by collagen fiber, but by basal cells, fibroblasts, red blood cells, capillary walls, or accumulating lipid pieces around the wall. The mean THG brightness of segmented collagen fiber in the modified ROI of each qualified subimage was recorded (${\mathrm{THG}}_{\text{Collagen}}$). For the part of basal cell cytoplasm, at least three qualified subimages were put into the program to segment basal cell cytoplasm based on THG information (Fig. 4). To deal with higher incidence of false segmentation due to low contrast of cells lacking melanin, manual removal of apparent noncell segmentation was applied. If after the removal there were fewer than 10 cells segmented, then pure manual selection of cytoplasm was applied for the subimage. The mean THG brightness of segmented cytoplasm of each qualified subimage was recorded (${\mathrm{THG}}_{\text{Cytoplasm}}$).

Finally, the intrinsic basal cell cytoplasm to collagen fiber THG-brightness ratio can be defined as follows:

$$\frac{{\mathrm{THG}}_{\text{Cytoplasm}}-\text{Noise}}{{\mathrm{THG}}_{\text{Collagen}}-\text{Noise}},$$

where noise is the mean THG brightness out of the rectangular ROI at the bottom 80th subimage in the stack. The ratio was calculated for each qualified subimage in a stack, and the final ratio obtained from the stack was the average of every subimage.

#### 2.3.5 Inspecting phase

According to the previous study on *in vivo* human THG microscopy, the cytoplasm of basal cells to collagen fiber THG-brightness ratio is about 1.6 at the dermal–epidermal junctions of the lightest phototype I skin.^8^ Although mostly such stacks were already ruled out during the previewing phase, stacks with the ratio higher than 1.6 were considered as melanin-containing and therefore unqualified for our intrinsic ratio study without melanin.

Following the above-mentioned phases, an intrinsic THG brightness ratio of 1.106±0.055 was obtained, with mean and standard error of the mean provided. This mean value was averaged from 14 stacks of nine patients with vitiligo, among them, there are six men and three women. Images were acquired at locations, such as right anterior chest wall, forehead, back, abdomen, shoulder, and junction of the shoulder and neck.

## 3 Applying THG Microscopy to Histologically Assess the Treatment of Vitiligo

**Fig. 5:**
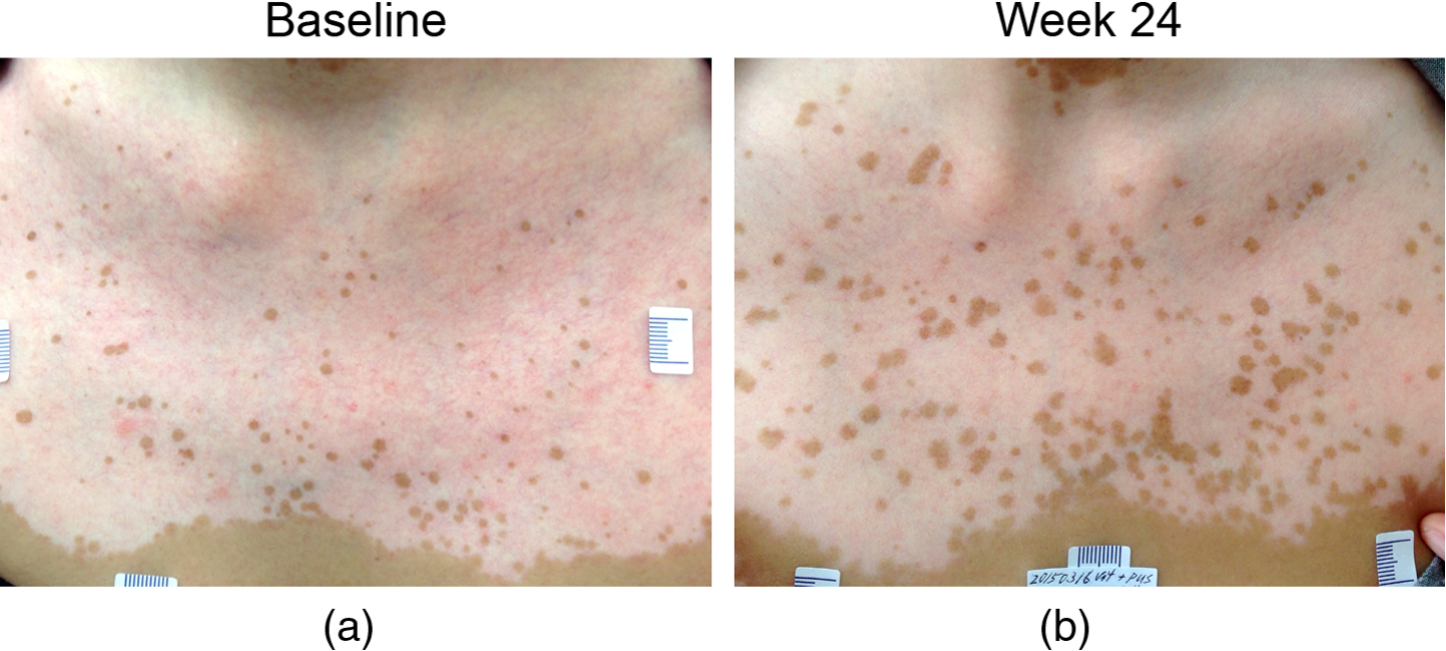
(a, b) Enhanced perifollicular repigmentation of vitiligo after 24-week NB-ultraviolet B (NB-UVB) treatment. PGA score 2 (perifollicular repigmentation with confluence <10%) was rated.

The low ratio value indicates the high sensitivity of THGM to observe minute changes in the appearance of melanin inside or around the basal cells. We thus further investigated the potential to apply THGM for the assistance of assessment in vitiligo treatments. The objective of the clinical study is to investigate the potential role of THGM to evaluate repigmentation in patients with vitiligo. A total of 23 patients having stable generalized vitiligo on the face or trunk were recruited. Subjects could receive treatments, including topical corticosteroids, calcineurin inhibitors, calcipotriol, and UVB phototherapy for their vitiligo. In each patient, one target vitiliginous lesion and one normal skin area away from vitiligo were selected, and the center of the selected areas was first imaged by HGM at baseline. At week 24, we evaluated the target lesion for the therapeutic efficacy based on the seven-point PGA score, as presented in Table 1. For instance, a 57-year-old female subject having scattered perifollicular repigmentation with confluence <10% after receiving 24-week NB-UVB treatment was scored as PGA 2 (Fig. 5). As a result, 15 of the 23 patients showed no improvement based on the PGA scores (with score 0, −1, or −2).

**Table 1:** The seven-point PGA score.

−2	Disease progression as patches
−1	Active border extension
0	No change
1	Round follicular repigmentation
2	Perifollicular repigmentation with confluence <10% of the lesion
3	Repigmentation between 10% and 90% of the lesion
4	Repigmentation with confluence >90% of the lesion

**Fig. 6:**
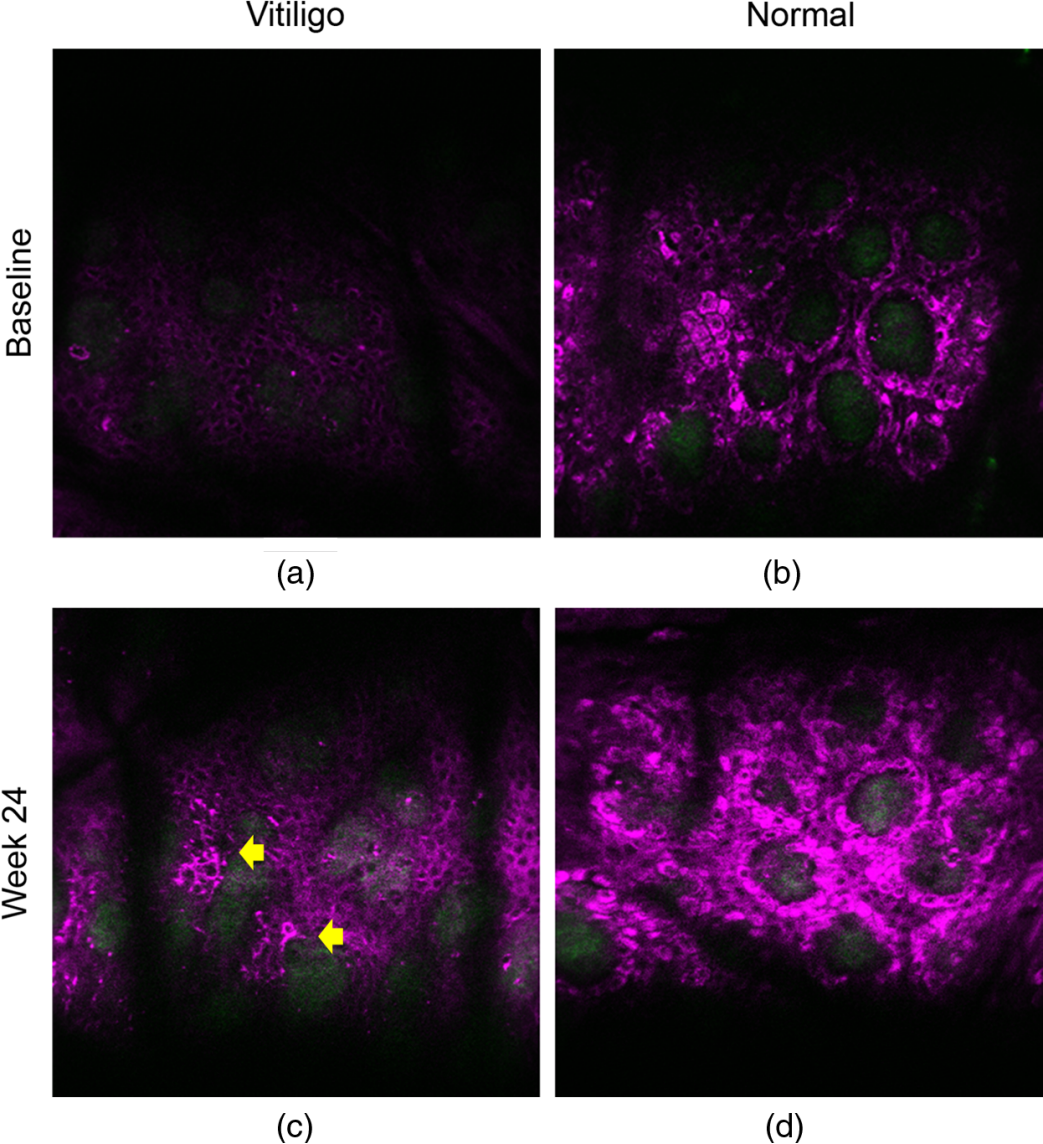
*In vivo* THG microscopy images with an *en face* view in a representative case with PGA score-1 at week 24. (a) The baseline images at the depth of basal cells showed low intensity of THG-brightness in the vitiligo lesion, compared to the normal skin (b). (c) Presence of THG-bright dendritic melanocytes (yellow arrows) and mildly increased THG-brightness in part of the basal and suprabasal keratinocytes after 24-week NB-UVB therapy. (d) The intensity of THG was also increased in normal basal cells after NB-UVB treatment. THG is shown in purple pseudocolor. Image size: $250\times 250\;\mu m^{2}$.

**Fig. 7:**
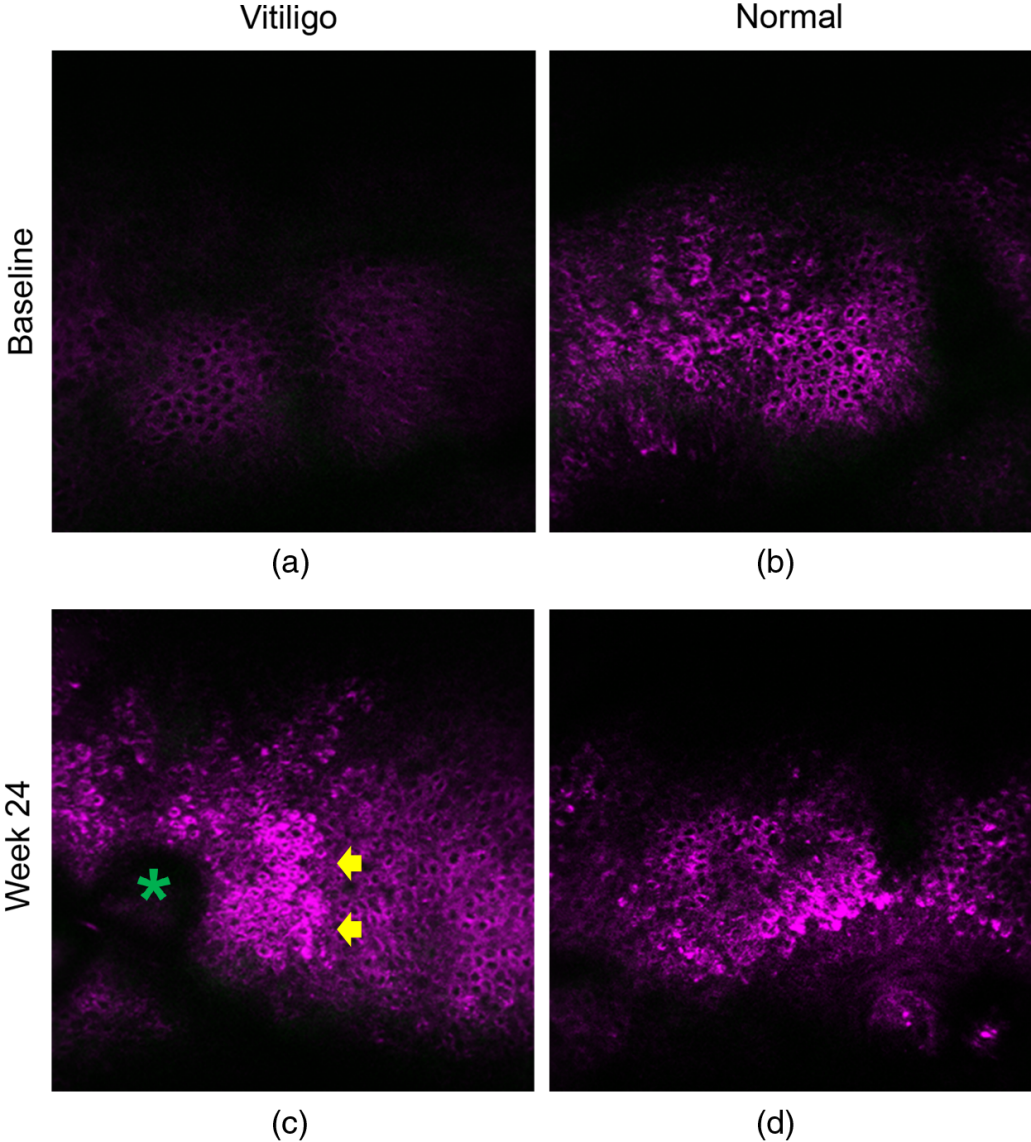
*In vivo* THG microscopy images with an *en face* view in a representative case with PGA score-1 at week 24. (a) The baseline images at the depth of basal cells showed low intensity of THG-brightness in the vitiligo lesion, compared to the normal skin (b). (c) Presence of THG-bright basal cells (yellow arrows) around the follicular space (green asterisk) in the ORS and surrounding basal keratinocytes was noted after 24-week NB-UVB therapy. The intensity of THG was compatible with the normal basal keratinocytes (d). THG is shown in purple pseudocolor. Image size: $250\times 250\;\mu m^{2}$.

To examine the potential of *in vivo* HGM on assessing the treatment efficacy of vitiligo, we further imaged by HGM the center of the target vitiligo lesions, which responded poorly to current treatments in these 15 patients at week 24. In a representative 41-year-old female patient having the target vitiligo lesion on the abdomen, the slide-free *en face* THGM images showed low THG intensity in the vitiligo lesion at baseline [Fig. 6(a)], compared to the normal basal cells [Fig. 6(b)]. At week 24, although being scored as PGA-1, the THGM clearly revealed the reappearance of dendritic melanocytes (yellow arrows) and repigmentation with increased THG brightness on part of the basal and suprabasal keratinocytes [Fig. 6(c)]. The surrounding normal skin also showed enhanced THG signals in the stratum basale [Fig. 6(d)], which possibly resulted from the tanning effect of UVB treatment. Figure 7 showed the representative THG imaging from a 55-year-old female patient who had the target vitiligo on the mandible and was scored as PGA-1 at week 24. At baseline, the basal cells in the target vitiligo lesion had very weak THG signals [Fig. 7(a)], compared with the normal skin [Fig. 7(b)]. After 24-week NB-UVB treatment, increased THG signals distributed in the ORS and the surrounding basal keratinocytes were noted, which indicated the early phenomenon of perifollicular repigmentation [Fig. 7(c)]. The intensity of THG in the repigmentation area was compatible with the normal basal keratinocytes [Fig. 7(d)]. These microscopic improvements were not assessable by the physician’s naked eyes. The relative dark THG background of vitiliginous basal cell cytoplasm lacking melanin provided an excellent signal-to-background ratio for the visualization of the repigmentation.

As a result, our THGM provides extra histopathological information to assist the evaluation of the therapeutic effect. Seven out of 15 patients showed improvement under UVB treatment based on the THGM images. It is noted that the pattern of the repigmentation as evaluated by physicians was follicular in all of the lesions. This indicates that melanocytes in the ORS or bulge of the hair follicle were stimulated by NB-UVB and caused follicular pigmentation in the vitiligo lesions.

## 4 Noninvasiveness and Photodamage Concern

The invasive nature of tightly focused femtosecond light is always a concern of clinical applications. With a tightly focused femtosecond laser light, the photodamage on skin can be roughly divided into the linear photothermal and photochemical effects and the nonlinear multiphoton-induced ionization and plasma effects. For 1260-nm light, the major linear absorbents in human skin will be water and lipid, and temperature rise would be the most likely effect. A recent metabolism study^17^ compared the effects of epidermal growth factor application and low-level near-infrared (NIR) laser therapy (LLLT, 780 nm, 25 mW, 0.5, 1.5, and $3\;J/{\mathrm{cm}}^{2}$) on cultured epithelial cells with three applications per day for 3 days. LLLT with a total $27\;J/{\mathrm{cm}}^{2}$ dosage was found to enhance cell migration; however, no significant effects of laser irradiation on other cell functions were observed. For multiphoton ionization effect with tightly focused NIR femtosecond light on a diffraction-limited spot under a high NA objective, live cells irradiated with 730 to 800 nm (1.7 to 1.55 eV) beams of >1  mW average power (total exposure per cell = 0.2 J) were found to inhibit cloning efficiency.^18^ A previous study of mammalian embryos^19^ indicated that by moving the tightly focused femtosecond excitation wavelength to 1047 nm (1.18 eV), with no more than 13-mW average power (five optical scanning sections collected every 15 min; total exposure=2  J/embryo), the imaged embryos were found to be able to maintain viability. The proportion of the embryos developed to morulae and blastocysts was not significantly different between imaged and nonimaged embryos (total exposure=2  J/embryo; n=21 embryos: imaged=0.89±0.15; nonimaged=0.83±0.24).^19^ This significant reduction on femtosecond laser damage indicates the dominant role of multiphoton process in the cell viability under tightly focused femtosecond NIR light. For example, a three-photon process with 730-nm light can generate the effect of 243 nm (5.1 eV) UV light^20^ to cause DNA damage. When the excitation photon energy is lowered by shifting the laser wavelength toward longer wavelength, the order number of nonlinear damage process increases, thus drastically increasing the nonlinear damage threshold.^21^ For 1047-nm light, it will require four-photon processes to generate the similar effect of 5-eV photon light. For 1260-nm (0.98 eV) light, the nonlinear process required to generate similar 5 eV effects will be a five-photon process, a less likely event under low pulse energy on the order of 1 nJ, which is our case. By shifting the femtosecond laser wavelength even further to 1230 nm,^8^ with 120-mW average power (3 min continuously per embryo; total exposure = 21.6 J), the HGM-imaged embryos under tightly focused light were also found to maintain viability. The blastocyst development rate of the HGM-imaged embryos with laser exposure is 94% (n=85 embryos: blastocyst stage = 80 embryos), and the rate of the embryos without laser radiation is the same 94% (n=80 embryos: blastocyst stage = 75 embryos). The proportion of the embryos developing to blastocysts showed no difference between imaged and nonimaged embryos, thus indicating no observable influence on DNA expression. This conclusion was supported by other 1230-nm based HGM studies of *in vivo* zebrafish embryos developments,^22^^,^^23^ where even after 100 mW and 20 h continuous observation (total exposure 7200  J/embryo), our reported technique induced no observable biodamages so that there is no reduction in cloning efficiency and each cell could function and divide normally to accomplish the whole development process, exactly the same as the control group. For small animal validation on the cell viability under high average-power femtosecond Cr:F laser illumination, after 150 mW, 3-h continuous observation under the THG microscope in the $80\times 80\;{\mathrm{mm}}^{2}$ area (1620 J total exposure with $2.5\times 10^{7}\;J/{\mathrm{cm}}^{2}$),^24^ the observed hamster buccal tissues were excised immediately. Histological examinations were then performed under a light microscope by an experienced pathologist. This procedure was repeated for three hamsters. The buccal squamous epithelium and subepithelial stroma were reported to be normal. No evidence of coagulation necrosis could be found. For human skin study, a previous study using femtosecond 750-nm light^20^ indicated a higher three-photon damage threshold when compared with the direct irradiation experiments on embryos.^18^

In this clinical study, the central wavelength of the femtosecond excitation light is 1260 nm, longer than the 1230 nm. With a 105-MHz repetition rate, 100-mW radiation corresponds to a pulse energy of 0.95 nJ. In a previous neuron activity recording imaging by using three-photon microscopy in brain,^25^ tightly focused 1300-nm light with 50-mW average power and 400-kHz repetition rate, corresponding to 125 nJ per pulse, was applied for continuous imaging with no reported damage in GFP-labeled animals. With less than 100 times pulse energy, if the nonlinear damage process is up to the fifth order, the nonlinear photodamage per pulse in our study will be >(100)^5^ times lower than this previous three-photon study and thus can be totally negligible, even after considering the fact that our system has 250 times more pulses per second. To compare with the previous embryo and mucosa studies at 1230 nm, in each imaged skin location, the total exposure time for acquiring one HGM image stack in this trial was around 28.6 to 35.7 s, corresponding to a total of 2.86 to 3.57  J/exposure. With much lower total laser exposure on a similar wavelength, we thus do not expect photodamages.

As a result, subjectively, the vitiligo patients do not feel any heat or discomfort on the 100 mW Cr:F laser-irradiated skin, and the dermatological skin examination immediately after HGM imaging reveals no erythema, pigmentation, crust, or vesicular formation. Objectively, during the course (less than 30 min) of real-time HGM imaging, there is no morphological alteration of epidermal cells or dermal structures, such as structural distortion or cellular burst. We have repeatedly performed imaging on the same cutaneous area, and the HGM morphology remains the same. We further conclude that there are no sensitive temperature changes above 42°C to 43°C to cause protein denaturation or noxious stimulation and thus would like to refer the HGM system as a noninvasive imaging system for vitiligo patients. Similar conclusions on the clinical study have been reported previously.^14^^,^^26^^,^^27^

## 5 Summary

Melanin is known to provide strong THG contrast in human skin. With a high concentration in the basal cell cytoplasm, THG contrast provided by melanin overshadows other THG sources in human skin studies. For better understanding of the THG signals in keratinocytes without the influence of melanin, an *in vivo* HGM study was first conducted on vitiliginous skin. As a result, the THG-brightness ratio between the melanin-lacking cytoplasm of basal cells and collagen fibers was measured to be 1.106 at the dermal–epidermal junctions of vitiliginous skin, indicating high sensitivity of THGM for the presence of melanin. We then applied HGM to assist the histopathological assessment of therapeutic efficacy of vitiligo. As a result, our study indicates the high potential of THGM to provide early and sensitive microscopic assessment on repigmentation treatment in vitiligo.
